# BRAZILIAN VERSION OF THE SHRINERS HOSPITAL UPPER EXTREMITY EVALUATION (SHUEE): TRANSLATION, CULTURAL ADAPTATION, AND EVALUATION OF PSYCHOMETRIC PROPERTIES

**DOI:** 10.1590/1984-0462/2020/38/2018328

**Published:** 2020-04-30

**Authors:** Renata D’Agostini Nicolini-Panisson, Ana Paula Tedesco, Jon Robert Davids, Lisa Vorpagel Wagner, Rita Mattiello, Márcio Vinícius Fagundes Donadio

**Affiliations:** a Pontifícia Universidade Católica do Rio Grande do Sul, Porto Alegre, RS, Brazil.; b Centro Universitário da Serra Gaúcha, Caxias do Sul, RS, Brazil.; c Instituto de Neuro-Ortopedia, Caxias do Sul, RS, Brazil.; d Shriners Hospital for Children, Greenville, South Carolina, United States of America.

**Keywords:** Cerebral palsy, Hemiplegia, Upper extremity, Hand, Validation studies, Paralisia cerebral, Hemiplegia, Extremidade superior, Mãos, Estudos de validação

## Abstract

**Objective:**

To validate the upper limb assessments tool, Shriners Hospital Upper Extremity Evaluation (SHUEE), for individuals with hemiplegic cerebral palsy in the Brazilian population.

**Methods:**

Validation study to translate and culturally adapt the Manual and the instrument. The psychometric properties evaluated were reliability and convergent validity. Reliability was determined by internal consistency (Cronbach’s α coefficient), ceiling and floor effect, sensitivity to changes, and intra- and interobserver agreement. Convergent validity was performed using the Pediatric Motor Activity Log, the self-care scale of the Pediatric Evaluation of Disability Inventory, and the Manual Ability Classification System.

**Results:**

We evaluated 21 individuals with hemiplegic cerebral palsy, with a mean age of 8.7±4.0 years. After the instrument was translated, there was no need for cultural adaptation. The total Cronbach’s α coefficient was 0.887 (95% confidence interval [95%CI] 0.745-0.970). We calculated sensitivity to changes in five subjects who underwent treatment with Botulinum Toxin Type A and physical therapy, with a significant difference between pre- and post-treatment evaluations in the Spontaneous Functional Analysis and Dynamic Positional Analysis. Convergent validity showed a significant correlation of the Spontaneous Functional Analysis and Dynamic Positional Analysis with the scales evaluated. All items of SHUEE presented high intra- and interobserver agreement.

**Conclusions:**

The results revealed that the Brazilian version of the SHUEE demonstrated good reliability and convergent validity, suggesting that it is an adequate and reliable tool for individuals with hemiplegic cerebral palsy in the Brazilian population.

## RESUMO

**Objetivo:**

Validar o instrumento de avaliação do membro superior, *Shriners Hospital Upper Extremity Evaluation* (SHUEE), para indivíduos com paralisia cerebral hemiplégica da população brasileira.

**Métodos:**

Estudo de validação no qual foi realizada tradução e adaptação cultural do manual e do instrumento. As propriedades psicométricas avaliadas foram confiabilidade e validade convergente. A confiabilidade foi determinada através da consistência interna (coeficiente α de Cronbach), efeito teto e chão, sensibilidade à mudança e concordância intra e interobservador. A validade convergente foi realizada utilizando-se o *Pediatric Motor Activity Log*, a escala de autocuidados do *Pediatric Evaluation of Disability Inventory* e o *Manual Ability Classification System*.

**Resultados:**

Foram avaliados 21 indivíduos com paralisia cerebral hemiplégica com idade média de 8,7±4,0 anos. Após a tradução do instrumento, não houve necessidade de adaptação cultural. O coeficiente α de Cronbach total foi de 0,887 (intervalo de confiança de 95% [IC95%] 0,745-0,970). A sensibilidade à mudança foi calculada em cinco indivíduos que realizaram aplicação de Toxina Botulínica tipo A e fisioterapia, apresentando diferença significativa entre a avaliação pré e pós-tratamento na Análise Funcional Espontânea e Análise Posicional Dinâmica. A validade convergente mostrou correlação significativa da Análise Funcional Espontânea e Análise Posicional Dinâmica com as escalas avaliadas. Todos os itens do SHUEE apresentaram concordâncias fortes, tanto na avaliação intra quanto na interobservador.

**Conclusões:**

A versão brasileira do SHUEE demonstrou um bom desempenho em relação à confiabilidade e validade convergente, sugerindo ser uma ferramenta adequada e confiável para os indivíduos com paralisia cerebral hemiplégica na população brasileira.

## INTRODUCTION

Individuals with hemiplegic cerebral palsy (CP) often present involvement of the upper limb (UL), leading to limitations of its functionality. The use of specific scales and evaluations helps health care professionals to analyze these limitations and plan the treatment. The literature reveals different methods to evaluate the UL in hemiplegic CP, including the Melbourne Assessment of Unilateral Upper Limb Function (MUUL), Quality of Upper Extremity Skills Test (QUEST), Assisting Hand Assessment (AHA), ABILHAND-Kids, and Shriners Hospital Upper Extremity Evaluation (SHUEE).^1^ ^,^ ^2^ However, most of the tests published take into greater consideration the capacity to perform a given task, measuring time to implement a particular function, than the way it is carried out, i.e., the main dynamic aspects.

In Brazil, the evaluation of the UL in hemiplegic CP is still limited, considering that no instruments to assess the capacity and performance of UL activity have been translated and validated for the Portuguese language. The only instruments validated for Brazilian Portuguese are the Pediatric Evaluation of Disability Inventory (PEDI),^3^ ^,^ ^4^ the Manual Ability Classification System (MACS),^5^ ^,^ ^6^ and more recently, the ABILHAND-Kids.^7^

SHUEE is an instrument that evaluates the UL in children and adolescents aged three to 18 years with hemiplegic CP.^8^ Differently from other tools, SHUEE not only assesses the individual’s capacity to use the UL for activities but measures the quality of the movement in UL segments and also guides treatment based on the final score.

The use of SHUEE is superior to conventional clinical evaluation when deciding on a treatment plan. One study assessed the agreement of therapeutic planning indicated after conventional evaluation with that proposed by SHUEE and found moderate agreement. That is, in 23.5% of cases, the treatment plan changed, either to add surgical procedures or substitute techniques. This finding shows the importance of using a quantitative evaluation and video-based instrument such as SHUEE to guide the therapeutic decision.^9^

Considering that SHUEE is a free assessment tool that enables a specific evaluation of the hemiplegic UL, discussing functional and positional aspects of the joints, and helping guide the treatment, the purpose of this study was to validate SHUEE for individuals with hemiplegic CP among the Brazilian population.

## METHOD

This is a validation study with two phases: phase 1 - translation and cultural adaptation, and phase 2 - validation of SHUEE for the Portuguese language.

Phase 1 - translation and cultural adaptation of SHUEE for the Portuguese language - followed the stages proposed by the American Academy of Orthopaedic Surgeons.^10^ Two independent translators, native speakers of Brazilian Portuguese (T1, T2), performed the English/Portuguese translation; one of them knew the concepts assessed by the instrument, while the other did not work in the health area. A committee composed of one specialized physical therapist, one neuro-orthopedist, and two translators compared and discussed these two translated versions. In case of divergences, the documents were adapted to elaborate a harmonized version, resulting in a single instrument in Portuguese (T12). Two independent translators, native English speakers (R1, R2), performed the back-translation of the harmonized Portuguese/English version. The translators were not familiar with the original version of the instrument and did not have a background in the health area related to the concepts explored by the instrument. The harmonization between both translators was done in the same way resulting in a single version in English (R12). Two researchers reviewed and compared the harmonizations (T12, R12) with the original in order to evaluate semantic and idiomatic equivalences; the original authors of the instrument performed the international harmonization, in which they assessed the versions resulting from the first and second harmonization. During this phase, the manual and forms of SHUEE were translated, as described above.

For phase 2, the sample was selected at a private rehabilitation clinic in the South of Brazil. The selection was performed using a database with 626 patients diagnosed with CP, 55 of them with a topographic classification of hemiplegia. The 21 patients included in phase 2 were randomly selected among the 55 hemiplegics. The exclusion criteria adopted were children and adolescents with clear cognitive limitations that would prevent them from performing the activities, besides those who had undergone a prior surgical procedure on the UL involved and/or application of Botulinum Toxin Type A (BTX-A) within less than 6 months. For a clinical characterization of the sample, participants were classified using the Gross Motor Function Classification System (GMFCS),^11^ ^,^ ^12^ ^,^ ^13^ MACS,^5^ ^,^ ^6^ Functional Mobility Scale (FMS),^14^ ^,^ ^15^ and with a hemiplegic gait pattern.^16^

The psychometric validation (phase 2) evaluated the following:

- *Reliability*, including the assessment of: a) internal consistency, using the Cronbach’s alpha coefficient (α-C); b) sensitivity to changes; and c) intra- and interobserver reliability. Sensitivity to changes was evaluated in all patients who underwent the treatment indicated by SHUEE, and, in these cases, a post-treatment SHUEE was performed two to four months later. For the inter- and intraobserver reliability, videos of ten randomly chosen patients (among the 21 of the total sample) were given to each of the five physical therapists who underwent training to use SHUEE and blindly scored the ten patients on two occasions, at a 2-week interval. Researcher RDNP administered the training to five physical therapists at the same time, with duration of six hours, corresponding to four hours of theory, providing information and examples of cases from the SHUEE Manual, and two hours of practical work with exercises on SHUEE scoring, using the case study video films supplied in the Manual.
- *Convergent validity*, which evaluated whether the measure of the SHUEE constructs is correlated with those of other equivalent instruments, demonstrating if the instrument is valid to assess what is intended. A single physical therapist (RDNP) performed and scored SHUEE and the other instruments (MACS, PEDI, Pediatric Upper Extremity Motor Activity - PMAL) for convergent validity in a sample of 21 children and adolescents. The sample was estimated based on the validation study of SHUEE^8^ and considering PEDI as a variable for convergent evaluation. We used a minimum detectable correlation of 0.57, with a standard deviation of 11.4 and 20.3, respectively, a power of 90%, and a level of significance of p<0.05.

SHUEE^8^ is a video-based evaluation that consists of two sessions. The first session analyzes ranges of passive and active movement, spasticity, history, and independence in the activities of daily living, besides the goals of patients and family. The second session uses films of 16 tasks and includes three components. Spontaneous Functional Analysis (SFA) evaluates the spontaneous use of the limb involved in nine common tasks, utilizing a modified House functional classification system.^17^ ^,^ ^18^ The dynamic positional analysis (DPA) assesses the segmental alignment of the extremity affected (thumb, fingers, wrist, forearm, and elbow) during the performance of 16 selected tasks. The final component, the grasp and release analysis (GRA), evaluates the subject’s capacity to hold an object with his or her fingers and release it with the wrist in the flexion, neutral, and extension position. SHUEE uses a numerical score, and the evaluations of SFA are scored on a scale from zero (does not use) to five (spontaneous use, partial to complete) with a maximum score of 45. Alignments of the DPA for each anatomical segment are scored on a scale from zero (pathological alignment) to three (normal or optimal alignment) with a maximum score of 72. The GRA is scored on a scale of zero (no - it was not possible to grasp and release) or one (yes - able to grasp and release) for each of the three wrist alignments (flexion, neutral, extension), with a maximum score of 6. For clinical use, the results are expressed as a percentage of the maximum score possible for each section.^8^

We performed the statistical analysis using the software *Statistical Package for the Social Sciences* (SPSS), version 20.0 (SPSS Inc., Chicago, IL). In all cases, the level of significance established was 5%. The normality of the data was verified with the Shapiro-Wilk test. Since the variables had a symmetrical distribution, data were expressed as mean and standard deviation. Categorical variables were presented as absolute and relative frequency. We evaluated reliability through internal consistency (α-C), sensitivity to changes (paired Student’s t-test), and inter- and intraobserver coefficient (intraclass correlation coefficient - ICC). The α-C values were considered adequate when ≥0.700. The proportion of individuals with a minimum (floor effect) and maximum score (ceiling effect) was also calculated. We determined the convergent validity by evaluating the possible correlations of SHUEE items (SFA and DPA) with PMAL, the self-care domain (functional skills scale and caregiver assistance scale) of PEDI, and MACS. Due to the quanti-qualitative characteristics of SHUEE, we used the Spearman’s rank correlation test, and classified the results according to the correlation coefficient (r) - very strong (r>0.9), strong (r from 0.7 to 0.9), moderate (r from 0.5 to 0.7), and weak (r from 0.3 to 0.5).

Regarding ethical aspects, one of the authors of the original article of SHUEE was contacted for permission to translate the tool into Brazilian Portuguese and validate it. After permission was given, the Research Ethics Committee of the Pontifícia Universidade Católica do Rio Grande do Sul approved the study. All the participants’ caregivers signed the informed consent form at the time of evaluation.

## RESULTS

In phase 1 -translation and cultural adaptation -, we found only grammatical and vocabulary discordances among the translators, which did not affect the semantic equivalence of the content. These disagreements were discussed and harmonized and, due to the practical content of the UL evaluation, there were no problems with idiomatic and cultural equivalence (colloquial expressions), so the cultural adaptation was not necessary.

Phase 2 - psychometric validation - involved a total of 22 individuals with hemiplegic CP. One individual was excluded because he had undergone previous orthopedic surgery on the UL. Thus, the final sample consisted of 21 children and adolescents. Table 1 presents the general characteristics of the sample studied.

**Table 1:** Demographic and clinical characteristics of children and adolescents with hemiplegic cerebral palsy.

Variables	Total sample (n=21)
Demographic characteristics	
Age, mean±SD	8.7±4.0
Male, n (%)	8 (38.1)
Right hemiplegia, n (%)	13 (61.9)
Left laterality, n (%)	13 (61.9)
Clinical characteristics	
GMFCS, n (%)	
I	15 (71.4)
II	6 (28.6)
MACS, n (%)	
I	6 (28.6)
II	8 (38.1)
III	7 (33.3)
Hemiplegic Gait Pattern, n (%)	
I	10 (47.6)
II	8 (38.1)
III	2 (9.5)
IV	1 (4.8)
FMS 5 meters, n (%)	
5	6 (28.6)
6	15 (71.4)
FMS 50 meters, n (%)	
5	7 (33.3)
6	14 (66.7)
FMS 500 meters, n (%)	
5	8 (38.1)
6	13 (61.9)

GMFCS: Gross Motor Function Classification System; MACS: Manual Ability Classification System; FMS: Functional Mobility Scale; SD: standard deviation.

Table 2 describes the psychometric characteristics of the Brazilian version of SHUEE.

**Table 2:** Psychometric characteristics of the Shriners Hospital Upper Extremity Evaluation in children and adolescents with hemiplegic cerebral palsy.

	Shriners Hospital Upper Extremity Evaluation		
	Spontaneous Functional Analysis	Dynamic Positional Analysis	Grasp and Release Analysis
Number of activities, n	9	16	6
Actual score (n=21), mean±SD	27.9±13.4	49.6±14.5	4.4±2.3
Floor effect, n (%)	0 (0)	0 (0)	3 (14.3)
Ceiling effect, n (%)	4 (19.1)	1 (4.8)	13 (61.9)
α-C, mean (95%CI)	0.988 (0.972-0.997)	0.777 (0.415-0.942)	0.933 (0.831-0.983)
α-C total= 0.842 (0.79-0.88)			

n=21. α-C: Cronbach’s alpha coefficient; CI: confidence interval; SHUEE: Shriners Hospital Upper Extremity Evaluation; SD: standard deviation.

### Reliability

In the analysis of internal consistency, the α-C of the total SHUEE (all items included) and of the DPA was good, with values of 0.887 (95% confidence interval [95%CI] 0.745‒0.970) and 0.777 (95%CI 0.415‒0.942), respectively. On the other hand, we obtained excellent results for SFA and GRA, with α-C of 0.988 (95%CI 0.972‒0.997) and 0.933 (95%CI 0.831‒0.983), respectively. The ceiling effect was observed in the three items of SHUEE. However, only GRA showed a floor effect.

Sensitivity to changes was evaluated in five individuals who had an indication for and underwent the application of Botulinum Toxin Type A (BTX-A) associated with physical therapy and guidance for home exercises. Table 3 shows the results of SHUEE performed before and after treatment. As expected, after treatment with BTX-A and physical therapy, the mean scores of SFA and DPA presented significant changes, showing that the use and positioning of the UL improved during the activities and that SHUEE was able to detect these changes. On the other hand, the mean GRA score had no significant changes.

**Table 3:** Sensitivity to changes of the Shriners Hospital Upper Extremity Evaluation after treatment with Botulinum Toxin and physical therapy.

Measures	Before* (n=5)	After* (n=5)	Difference*	p-value
Spontaneous Functional Analysis	19.2±8.2 [10-31]	22.8±10.2 [12-36]	3.6±4.0 [2-9]	0.02
Dynamic Positional Analysis	42.4±11.2 [25-53]	53.6±7.2 [45-63]	11.2±6.30 [3-20]	0.01
Grasp and Release Analysis	4.2±1.8 [2-6]	4.4±1.7 [2-6]	0.2±0.5 [0-1]	0.37

*Data expressed as mean±standard deviation, with the minimum and maximum values in brackets.

Table 4 presents the results of the evaluation of inter- and intraobserver reliability of SFA, DPA, and GRA. The findings show a strong inter- and intraobserver reliability.

**Table 4:** Intraobserver and interobserver reliability of the Brazilian version of the Shriners Hospital Upper Extremity Evaluation.

Measures	Intraobserver ICC (First x second evaluation)	Interobserver ICC (5 physical therapists)
Spontaneous Functional Analysis	0.997 (0.996-0.998)	0.996 (0.990-1.00)
Dynamic Positional Analysis	0.990 (0.979-0.998)	0.987 (0.979-0.993)
Thumb	0.964 (0.944-0.986)	0.986 (0.976-1.00)
Fingers	0.994 (0.984-1.00)	0.994 (0.984-1.00)
Wrist	0.957 (0.922-0.989)	0.929 (0.887-0.959)
Forearm	0.995 (0.993-0.999)	0.996 (0.977-1.00)
Elbow	0.956 (0.889-1.00)	1.00 (1.00-1.00)
Grasp and Release Analysis	1.00 (1.00-1.00)	1.00 (1.00-1.00)

*Values expressed as mean ICC (minimum-maximum); ICC: intraclass correlation coefficient; n=10 (videos of 10 patients were blindly scored for 5 physical therapists on two occasions, with a 2-week interval).

### Convergent validity

SFA was moderately correlated with the self-care domain of part I of PEDI - functional skills scale (r=0.68; p=0.00) - and with MACS (r=-0.68; p=0.00); however, it was inverse in the latter. Furthermore, we found a strong correlation with the scales of frequency (r=0.86; p=0.00), quality (r=0.86; p=0.00), and spontaneity (r=0.80; p=0.00) of PMAL; and with the self-care domain of part II of PEDI - caregiver assistance scale (r=0.75; p=0.00). In contrast, DPA showed a moderate correlation with parts I - functional skills scale (r=0.62; p=0.00) - and II - caregiver assistance scale (r=0.63; p=0.00) - of the self-care domain of PEDI and inverse with MACS (r=-0.54; p=0.01). We identified a strong correlation with the PMAL scales of frequency (r=0.83; p=0.00), quality (r=0.83; p=0.00), and spontaneity (r=0.76; p=0.00). This finding indicates that the greater the spontaneous use of the UL and its positioning in functional activities, the better the manual skill, the greater the functional skill for self-care, the less assistance is necessary from the caregiver for the self-care activities, and the greater the frequency, quality, and spontaneity of the use of the hemiplegic UL in everyday activities.

## DISCUSSION

The Brazilian version of SHUEE showed a good psychometric performance, suggesting that it is a useful tool in the Brazilian cultural context. Moreover, our study appears to be the first to test the internal consistency of SHUEE in the reliability analysis by calculating the α-C. The results showed a good α-C value for the evaluation of all items of SHUEE and DPA, besides excellent values for SFA and GRA. Thus, SHUEE proved to be valid and reliable for SFA, DPA, and GRA in children and adolescents with hemiplegic CP, with values similar to those of the original findings of the instrument.

Another reliability analysis performed in our study was the sensitivity of the instrument to detect changes, with both SFA and DPA showing significant differences after treatment with BTX-A and physical therapy, demonstrating that SHUEE presented sensitivity to detect this change. As evidenced in the literature, treatment with BTX-A can improve the position of UL segments while a given function is performed.^19^ However, few patients in the present study sample underwent this treatment, resulting in a relatively small sample to effectively identify the sensitivity to changes of SHUEE. The original study of SHUEE also showed interesting results on this subject, with significant alterations in these two items of SHUEE after the surgical treatment that transferred the ulnar flexor tendon from the carpus to the short radial extensor of the carpus in eighteen individuals with hemiplegic CP.^8^

Our excellent ICC findings, detected in the analysis of inter- and intraobserver reliability, for SFA and DPA were similar to those of the original study of SHUEE, as well as the 100% intra- and interobserver concordance in both studies for GRA.^8^ Furthermore, our study and the original one^8^ presented the reliability analysis of all segments that form the DPA score and showed excellent ICCs for the thumb, fingers, wrist, and forearm segments, besides a 100% concordance in the elbow segment (interobserver). The physical therapists who participated in the evaluation of intra- and interobserver concordance in our study have one to ten years of clinical experience. These professionals completed standard training based on the SHUEE Manual, which included learning the assessment instrument and watching the key videos of interpretation and practicing videos of clinical cases. These results show that, after standard training using the SHUEE Manual, the instrument is reliable for clinical and scientific analysis of the UL in patients with hemiplegic CP.

Although the three-dimensional analysis of movement is standardized for the evaluation of lower limbs,^20^ its use for the UL is still a challenge, given the complexity of their movements.^21^ Thus, the convergent validity presented in our study was performed using the MACS and PEDI (self-care domain for the functional skills and caregiver assistance scales) instruments, through their correlations with two items of SHUEE: SFA and DPA. As shown in the original article on SHUEE,^8^ we also identified a moderate correlation of SFA with the self-care domain of PEDI. We further found moderate and strong correlations with the PMAL questionnaire and MACS. However, our study appears to be the first to correlate these instruments with SHUEE, which makes it difficult to discuss these results. PMAL, similarly to SHUEE, is a specific instrument for hemiplegic CP and assesses both the frequency and quality of use of the hemiplegic UL in activities, as reported by the child’s parents or guardians.^22^ MACS evaluates how the children with CP use their hands to manipulate objects at home, at school, or in the community^5^ ^,^ ^6^ and has been widely used as a classification system in research on the UL in CP.^23^

Frequently, the evaluation of the UL is based on the report of functionality from the caregiver or the patient, and on the physical examination. Nonetheless, the functional aspects, in which the limitations caused by the deformities become clearer, are rarely analyzed objectively. SHUEE proposes combining this usual evaluation with a video-based assessment that covers the functional analysis and the positioning of the segments during the activity. From this perspective, SHUEE has been used in the literature to demonstrate the results after surgical^24^ ^,^ ^25^ and conservative treatments, such as the use of BTX-A^24^ and Constraint-Induced Movement Therapy,^26^ in children and adolescents with unilateral CP. SHUEE has been used in Brazil and seems to help in clinical reasoning and decision-making process, besides enabling the formal measurement of the results of the therapies employed.^9^ ^,^ ^25^ ^,^ ^27^

The fact that the sample calculation was estimated from the analysis of convergent validity and based on the original study of SHUEE may constitute a limitation of this study. However, no previous studies have calculated the α-C for SHUEE, which would enable the sample calculation based on this item. Also, the instruments correlated with SHUEE in our study for convergent validity are based on the report of the caregiver’s perception regarding the functionality of the children, and not on another technical evaluation. Nevertheless, no instruments for the technical assessment of the UL of individuals with CP have been translated, culturally adapted, and validated for the Brazilian population. Moreover, future studies should consider factorial analysis to validate the construct and reproducibility of SHUEE.

In conclusion, the Brazilian version of SHUEE performed well in the psychometric properties evaluated: internal consistency, intra- and interobserver reliability, and convergent validity. Additional studies could contribute to analyze further the sensitivity to changes of the instrument. Thus, this version of SHUEE seems adequate and applicable to individuals with hemiplegic CP among the Brazilian population. The instrument is available for use upon request to the authors.
